# Review Department

**Published:** 1880-01

**Authors:** 


### REVIEW DEPARTMENT.

I.

Department of Agriculture. Special Report, No. XII. Investigation of Diseases of Swine, and Infectious and Contagious Diseases incident to other classes of domesticated animals. Washington Government Printing Office, 1879. 288 pages.

This interesting and valuable document owes its appearance to the action of the Commissioner of Agriculture, Wm. G. LeDuc, appointing several prominent veterinarians and physicians to examine into the prevalence of the swine-plague, chiefly in the Western States, and from which immense losses had occurred during the year 1877.

It contains an introductory which summarizes the scope of the work, and special reports of nine investigators, three of whom, Drs. Detmers, Law and Salmon, are veterinarians, the remainder, human physicians.

The remainder is taken up by correspondence relating to the prevalence of various diseases among domesticated animals; and four articles, respectively treating of Pleuropneumonia, a strange disease among North Carolina Cattle, the Cattle Plague and Glanders.

Dr. Detmers, V. S., besides detailing the well known symptoms and pathology of the swine-plague, more properly, we think, termed pig-typhoid, (Osler,) has made researches on the blood, demonstrating the existence, as he states, of a peculiar bacillus, the *b. suis*. His report is characterized by thoroughness and attention to details, and a series of experiments undertaken with Dr. F. W. Prentice of the Veterinary Department of the Illinois Industrial University, demonstrated that the *bacilli* occupied a causal relation to the disease. The first plate presents a very faithful colored lithograph of the lung in this affection, the second plate is not as clear as migbt be desired. A fine series of five colored plates exhibit the changes in the follicles of the intestines which, from their striking analogy to the corresponding changes in human typhoid, have given rise to one of the designations of the disease. The last plate represents outline drawings of *bacilli* in different situations and stages of developement.

Dr. Law follows with a report on the nature and vitality of the virus of this disease. He states the average period of incubation to be from three to seven days after inocculation, though it may last as long as fourteen. He endorses the opinion of Dr. Klein that the intestinal ulcers are not of the lymphoid (solitary and peyerian) glands, but of Lieberkuhn's follicles. The lithographs accompanying this part of the report are very obscure. The assumed relation of intestinal and other parasites is the next subject of Dr. Law's studies. He properly denies any relation between parasites and specific fever, but admits that the ravages of *strongyli*, especially when coincidently occurring in large herds, may lead to outbreaks simulating the so-called hog-cholera.

Excellent figures of these parasites are given.

Bisulphite of soda, carbolic acid, sulphate of iron, chlorate of lime, permanganate of potash, and bromide of ammonium failed to render the virus employed in inoculation harmless; chloride of zinc was, however, of marked efficiency in this direction.

The author recommends the stamping out process as the only efficient preventive of disastrous epidemics.

Dr. Voyles, who conducted his examinations in Indiana, considers what, from his description, seems to us to have been swine-fever, as a catarrhal pneumonia. He makes some very sound strictures on the unsanitary conditions in which swine are kept. Dr. Salmon makes similar observations in North Carolina, and endorses the stamping out process.

Dr. Dunlap of Iowa, in the absence of anything definite that he can find in the literature at his disposal, comes quite close to a proper nomenclature, by naming the disease: Typhus. He makes some statements on preventing the great susceptibilty of swine to epidemics, by due attention to a natural hygiene, which deserves heeding at the hands of stock raisers. Dr. Dyer, of Illinois, in all essential particulars, agrees with the preceding, as do also Drs. McNut and Hines, of Missouri and Kansas. An elaborate article on Glanders well illustrated by lithocaustic plates and written by Dr. Detmers, of Chicago, closes the volume.

He classifies Glanders as 1. Nasal glanders, the common form characterized by the peculiar nasal discharge, which Dr. Detmers states to be although the most conspicuous yet the least diagnostically valuable symptom. The principal symptoms are the swelling of the lymphatic glands and the ulcers on the nose. 2 Pulmonary glanders, 3. External glanders, so-called Farcy, of which with Gerlach he discriminates two forms, the subcutaneous or common farcy, and the *exanthematous* or skin farcy. After a very clear and sound exposé of the pathology of glanders in which Dr. Detmers follows the German pathologists he closes this chapter with the following passage, which we can only endorse to its fullest extent.

"The only rational treatment of a horse or other animal, affected with glanders, consists in a proper and effective application in the right place, of either half an ounce of lead or five inches of steel; and until such treatment is invariably adopted, or made compulsory, there will be no prospect whatever of freeing this country from this loathsome disease, dangerous even to man, in whom, if once infected, it is just as incurable as in horses."

This volume is on the whole a credit to its contributors and the government officers who urged the undertaking of the work. ***

II.

The Lung-Plague of Cattle, Contagious Pleuro-Pneumonia, by James Law, Professor of Veterinary Medicine, Cornell Uuiversity, with Illustrations: Ithaca, published by the author, 1879; 97 pages

Professor Law in this little volume goes over, in a clear familiar way, the main points in the history, symptoms, pathology, treatment and sanitary bearing of contagious pleuro-pneumonia. Although we may presume that most of onr readers are familiar with the subject and aware of the grave danger to our stock-interests which seems to have been successfully warded off by Professor Law and his associates, it may not be out of place to present some of that writer's results in abstract. Professor Law considers "pleuro-pneumonia" a misleading term, and seeks in the English equivalent of "Lungenseuche," *Lung plague of cattle*, a fitter title. The disease was conveyed to America (Brooklyn) in 1843 and millions have been lost in consequence of the importation of a single cow in that year. He holds that it never arises in this country except as the result of contagion and can be transmitted through mediate as well as through immediate contagion. The recovered animal may retain the power of infecting other cattle for even *twelve* or *fifteen* months, owing to the continued exhalation of late liquifying masses in the lungs. In summer the susceptibility is greater and the mortality higher and earlier. The advice to stock-raisers culminates in the dictum "breed your own cattle." He urges the purchasing of cattle only from places with a clear bill of health and transportation along safe roads only, and to quarantine them on arrival.

As prophylactics in exposed heads, the daily use of two drachms (7.50 gr) of sulphate of iron combined with half that quantity of carbolic acid is recommended. He condemns inoculation, and the passing of young cattle through the plague in order to eradicate the susceptibility of the disease, and recommends that diseased animals be slaughtered under the eye of an inspector, their hides slashed, and their carcasses deeply buried in a secluded place.

In warm and convincing language he points out that the stamping out of this dangerous affection permanently can only be effected by the Government of the United States. The little volume deserves perusal, not only at the hands of the veterinaaian, but also of every intelligent stock raiser. ***

